# Three-Dimensional Cell Culture Models to Study Respiratory Virus Infections Including COVID-19

**DOI:** 10.3390/biomimetics7010003

**Published:** 2021-12-25

**Authors:** Aya Harb, Mohammad Fakhreddine, Hassan Zaraket, Fatima A. Saleh

**Affiliations:** 1 Department of Experimental Pathology, Immunology & Microbiology, Faculty of Medicine, American University of Beirut, Beirut 11-0236, Lebanon; harb.aya.14@gmail.com (A.H.); hz34@aub.edu.lb (H.Z.); 2 Faculty of Medicine, American University of Beirut, Beirut 11-0236, Lebanon; maf54@mail.aub.edu; 3 Center for Infectious Diseases Research, Faculty of Medicine, American University of Beirut, Beirut 11-0236, Lebanon; 4 Department of Medical Laboratory Sciences, Faculty of Health Sciences, Beirut Arab University, Beirut 11-5020, Lebanon

**Keywords:** 3D culture models, spheroids, respiratory viruses, coronaviruses, COVID-19

## Abstract

Respiratory viral infections, including severe acute respiratory syndrome coronavirus 2 (SARS-CoV-2), are among the most common illnesses and a leading cause of morbidity and mortality worldwide. Due to the severe effects on health, the need of new tools to study the pathogenesis of respiratory viruses as well as to test for new antiviral drugs and vaccines is urgent. In vitro culture model systems, such as three-dimensional (3D) cultures, are emerging as a desirable approach to understand the virus host interactions and to identify novel therapeutic agents. In the first part of the article, we address the various scaffold-free and scaffold-based 3D culture models such as hydrogels, bioreactors, spheroids and 3D bioprinting as well as present their properties and advantages over conventional 2D methods. Then, we review the 3D models that have been used to study the most common respiratory viruses including influenza, parainfluenza, respiratory syncytial virus (RSV) and coronaviruses. Herein, we also explain how 3D models have been applied to understand the novel SARS-CoV-2 infectivity and to develop potential therapies.

## 1. Introduction

Understanding host responses to microorganisms as well as the virulence traits employed during host-pathogen interactions are key for drug design and development. Currently, the majority of our knowledge and understanding of the mechanisms of viral pathogenicity and the host response to infection is based upon studies that have been carried out using traditional 2D methods, with cells grown on flat plastic dishes. Although these simplified culture systems are essential to gain an insight into the fundamentals of host-pathogen interactions, cells in 2D are not exposed to the same conditions as cells in 3D tissues in the body and are therefore a poor representation of the in vivo microenvironment [1]. Current 2D cell culture methods lack the complex biological processes that occur in tissues in vivo [2,3,4,5]. Consequently, the analyzes of infectious diseases and their pathogenic mechanisms in these flat cultures may be misleading, even contradictory, therefore restricting therapeutic implementation which might explain the unsuccessful attempts to develop effective therapeutic drugs and vaccines [5,6].

In an attempt to recreate the complex microenvironment that pathogenic microorganisms encounter in the host tissues they infect, and for a more comprehensive understanding of host–pathogen interactions, the use of 3D culture systems is gaining increasing attention. 3D culture models will promote direct cell-cell contact, interactions of cells with the ECM and in-vivo like exchange of soluble factors; thus, allowing cells to more closely resemble the in vivo parental tissue than did their 2D monolayer counterparts. Thus, data gained from 3D in vitro methods can help to bridge the gap between conventional 2D culture studies and in vivo preclinical animal models. Moreover, 3D models can serve as a better platform for drug and vaccine development [7,8,9,10]. In this review, we present the various scaffold-free and scaffold-based 3D culture models such as hydrogel, bioreactors and hanging drops as well as present their properties and advantages over traditional 2D methods. However, tissues such as airways are usually in layers rather than a packed dense tissue; hence, 3D bioprinting technology which involves layer-by-layer deposition of biologically-derived materials or cells was also discussed. Moreover, the review will cover the progress made on respiratory viruses using 3D culture methods such as influenza virus, parainfluenza virus, respiratory syncytial virus (RSV) and coronaviruses with special focus on severe acute respiratory syndrome coronavirus 2 (SARS-CoV-2).

## 2. Three-Dimensional (3D) In Vitro Models

As the notion of the 3D cell culture became more familiar in in vitro studies, and as the inter-disciplinary studies between cell biology and the biophysical sciences evolved along with the advances in technology, attempts have been made to establish high standard spheroid generating techniques. These techniques need to be efficient, quick, reproducible, practical, and ensure spheroids’ uniformity in size, limit cellular damage and cytotoxicity while promoting cell growth, shape and physiology. Methods to generate 3D cultures can be classified into scaffold-free and scaffold-based culture systems. While currently there is no optimal method that satisfies all research requirements, researchers can choose the most appropriate technique that aligns with their needs, where each method has its advantages and disadvantages [11]. Nonetheless, all methods generate 3D spheroids with increased expression of adhesion molecules, enhanced cell-cell communication, increased intercellular signaling and higher differentiation potential. All these factors allow 3D cultures to mimic the in vivo microenvironment more closely than the 2D cell culture systems [12,13,14]. Below we address the main properties and differences between the various scaffold-free and scaffold-based techniques.

### 2.1. Scaffold-Based 3D Cultures

In scaffold-based cultures, cell suspensions are seeded on matrices that serve as scaffolds on which the cells adhere to, and which dictate the three-dimensional shape of the obtained cell culture. These scaffolds can be composed from either natural or synthetic materials, which often raise the concern of biocompatibility [15].

#### Hydrogels

Hydrogels are 3D hydrophilic extracellular matrix (ECM)-rich meshes that contain a diverse selection of biopolymers and are commonly used as scaffold frameworks to surround and encapsulate cells [16]. These hydrogels’ properties mimic the ECM bed on which cells lay in vivo and which is crucial for cellular proliferation, survival, differentiation, polarization, and signal transduction [17,18]. The hydrogels’ hydrophilic nature, chemical stability, biological compatibility and biodegradability potential have made it a suitable model for 3D cell culture studies. It has offered a better representation of events occurring in vivo and consequently been used to bring deep insight into the viral infection process by investigating how virions diffuse in the ECM before attaching to cells [19,20]. Moreover, hydrogels have been ideal to provide a 3D environment for studying cell responses to viral infections as well as drug screening.

In a preliminary study by Suzuki and colleagues, human bronchial 3D cultures were successfully generated by embedding cryopreserved human bronchial epithelial cells in Matrigel, a natural ECM environment hydrogel [21]. This 3D model was then infected with SARS-CoV-2 with increased expression of the SARS-CoV receptor ACE2 (Angiotensin-converting enzyme 2) and TMPRSS2 (transmembrane serine proteinase 2), which is essential for the viral spike (S) protein priming [21]. Similarly, Han et al. reported that human lung 3D cultures generated from embryonic stem cells embedded in Matrigel, were permissive to SARS-CoV-2 infection [22]. Moreover, both studies demonstrated that these 3D culture models can be used to evaluate antiviral effects of candidate compounds against SARS-CoV-2 [21,22].

This proves that generation of human lung 3D cultures using Matrigel can mimic the lung function in vivo and thus be used be a useful model to study viral infections including SARS-CoV-2 [23].

### 2.2. Non-Scaffold 3D Cultures

Scaffold-free 3D cultures rely on stationary or rotary forces to aggregate cells found in suspension into spheroids. Accordingly, they are divided into static and dynamic systems. One feature that distinguishes the non-scaffold cultures is the predominance of cell-to-cell interactions rather than cell-to-ECM thus allowing the natural aggregation and assembly of cells and the development of spheroids similar to the development seen in vivo during organ formation [24,25].

#### 2.2.1. Bioreactors (Dynamic)

Bioreactors or rotating wall vessels (RWV) are instruments that generate 3D spheroids by creating a microgravity environment. It is based on rotational motions which keep the cells floating in suspension while allowing them to aggregate into spheroids [26]. The system is driven by a motor which continuously revolves a pillar around an x-axis while being connected to culture chambers having the cell suspensions [26]. Consequently, the cells are maintained at a constant state of free-fall [6,26]. These chambers accommodating the cells are incubated at 5% CO_2_, humidified incubator. When the bioreactor is set up to begin cell incubation, it starts off at a slow rate of around 15 RPMs. As the cell aggregates start to grow with increase in size, the rotation speed is progressively increased to maintain the aggregate in a free fall state [27]. Furthermore, for long-term experiments media change is made possible through the injection port of the culture chambers using a syringe.

Various primary cells and cell lines have been shown to form spheroids successfully in bioreactors [4,28,29]. Goodwin et al. reported the formation of 3D lung tissue-like assemblies from a combination of primary human bronchio-tracheal cells and human bronchial epithelial cell line using a RWV bioreactor [30]. Interestingly, these tissue-like assemblies showed properties similar to their native counterparts. Moreover, they were able to be infected with various respiratory viruses such as human parainfluenza virus type 3, RSV and SARS corona virus and thus can be used to study viral infections and virus-host interactions in a more physiological setting [30].

Today, rotating wall bioreactors proved to be successful in providing a low shear environment limiting cell damage and enabling long-term culture periods. In spite of this advancement, bioreactors have some limitations such as inconsistency in spheroid size and difficulty in monitoring the aggregates in real-time due to the continuous rotatory culture system [31]

#### 2.2.2. Spinner Flasks (Dynamic)

Spinner flasks produce spheroids by maintaining cells in suspension with continuous rotary motion that causes spontaneous cell collision and adhesion [32]. However, this system is distinctive for its internal impeller or magnetic stir bar which allows the continuous stirring of the suspended cells [32,33,34]. A balanced rotational speed is key for preventing the aggregates from settling at the flask bottom while minimizing harmful fluid shear stress caused by high rate of stirring. Additionally, the generated spheroids’ diameter can be managed by modifying the spinning speed, culture duration, culture medium composition and cell-seeding density [33]. The continuous fluid motion promotes gas and nutrient transport within the formed spheroids which can be composed of primary cells, cell lines or a fusion of different cell types [33].

#### 2.2.3. Hanging Drops (Static)

Hanging drop is the first recorded method for generating 3D aggregates where it was employed to create embryoid bodies [35]. It is based on the simple physics concepts of gravity and surface tension [35]. Culture medium containing cells at various seeding densities are pipetted as droplets on the surface of a tissue culture plate cover which is incubated upside down causing the droplets to suspend from the surface and cells to self-aggregate at the air-liquid interface thus forming a single spheroid per droplet [36]. Hanging drops are suitable to culture both primary cells and cell lines, in addition to co-cultures of different cell types forming heterotypic spheroids [37,38,39,40]. Despite hanging drops being used to generate spheroids of limited size range, the size of the spheroid can be adjusted by controlling the seeding density and incubation time [36]. While conventional hanging drop cultures require basic laboratory equipment and are easily monitored microscopically, they yield a limited number of spheroids and are tedious due to the difficulty in managing medium exchange and drug administration in such small volumes [41]. Today automated commercialized 96- and 384-well hanging drop arrays are available that help controlling spheroid size and facilitate hanging drop technique in terms of effort, reproducibility, cost and high-throughput screening capacity [42,43].

#### 2.2.4. Ultra-Low Attachment Plates (Static)

One of the most common methods for spheroid generation is the use of round-bottom ultra-low attachment 96-well plates. The bottom of these ultra-low attachment 96-well plates are covered with non-adhesive materials, which inhibit cells from attaching to the plasticware [44]. The cells aggregate at the bottom of the ultra-low attachment wells after being dispersed in single cell suspensions of culture medium with or without methylcellulose [44,45]. A review of the literature shows that ultra-low adhesion plates have been used to culture a wide variety of cells including cocultures [46]. We have recently shown that A549 cells can be grown as spheroids in an ultra-low attachment 96-well plate to study RSV pathogenesis [47].

Undoubtedly, this method of generating spheroids is easy, simple, affordable and reproducible, where it allows the production of consistent spheroids of uniform size and shape [44]. Moreover, the cultures are easily observed and monitored since the plate material resembles any other laboratory polystyrene dish allowing microscopic observation. In addition, these 96-well plates are suitable for high throughput screening [46].

#### 2.2.5. Methods Using External Force (Electric Fields, Magnetic Force, and Ultrasound)

There are other systems similar to the centrifugation pellet culture that rely on external forces to obtain spheroids from cell floating in suspension. These systems can utilize electric fields, magnetic fields or ultrasound forces to assemble cell suspensions into spheroids. When electric fields are utilized, cell monolayers cultured in iso-osmotic solutions migrate and aggregate into 3D aggregates through dielectrophoresis which facilitate actin filament turnover [48]; whereas in ultrasound spheroid formation, an ultrasonic standing wave trap is used to cluster the cells [49]. As for magnetic fields, this method if performed by ensuring that magnetic cationic liposomes (MCLs) are endocytosed by cells which are then directed and collected over a magnetic field [24,50]. In 2013, a research group led by Glauco Souza created a 3D bronchiole coculture model from four human cell types using magnetic levitation that was found to mimic pulmonary bronchioles in vivo [51].

These techniques are run under strictly managed settings to minimize cellular damage; however, the physiological impact that these techniques have on cells needs further investigation. Moreover, these methods provide limited ability to regulate the spheroids’ sizes and require specific equipment, which limit the utilization of these methods.

#### 2.2.6. Microwell Arrays

Microwell arrays are a newly evolving method for spheroid generation which originated from developing spheroids in round-bottom ultra-low attachment 96-well plates [52]. It relies on imprinting microwells on non-adhesive substance such as agarose or polyethylene glycol (PEG) through micro-patterning techniques through the use of stamps. The resulting microstructures are of a specific shape and size which dictate the spheroids’ dimensions [53]. Single cell suspensions are loaded to the common channel after which the plate is slightly centrifuged in order to homogenously dispense the cells across the compartments [52,53]. This process further ensures uniformity of the produced spheroids’ size, and the identical shapes of the wells generate spheroids of uniform shape [7,17]. Such microwell structures are simple, economical, and can be performed in technologically modest laboratories. Moreover, they are compatible for high throughput drug screening and can be incorporated for use with microfluid systems [54,55].

#### 2.2.7. Microfluidic-Based Methods

A new technology named “microfuidics” has been developed in recent years in which cell suspensions are ran through a microchannel system and divided upon microwells which house these cells and the resulting spheroids [56]. The microwells trap the cells and enhance their aggregation through the micro-rotational movement of the medium which is induced by small bioreactors. These microwells are microfabricated on a silicon base which are covered with the microchannel system [57]. Additionally, some microfluidic platforms utilize non-adherent materials when designing the microwells to prevent spheroids from attaching. Poly(dimethylsiloxane) or PDMS is the most commonly used polymer for building the microchannels of the microfluidic platforms, since it is resistant to corrosion and mechanical changes which may result from pressure or temperature changes or contact with the culture medium [56]. These PDMS channels have inlets through which the cell suspension is deposited after which it is pumped through the system [56]. As for the cell suspension, cells can be either suspended in gelatinous hydrogel or in regular cell culture medium before being dispensed into the system.

Microfluidic systems produce a high yield of identically sized spheroids in a limited amount of time and devoid of extensive labor [56,58]. They are compatible for use in high throughput screening and analysis and in combination with advances machines where they can be supplied with biosensors for immediate observation and imaging [59]. Various cell types showed to successfully aggregate into spheroids in this system and co-cultures of different cell types were also possible [56,60,61].

Using microfluidic technology, organs-on-chips were developed to reconstitute the physiological and functional complexity of organs and thus mimic the in vivo microenvironment and present it in vitro on miniaturized platforms [62]. One of the most prominent developments, is the lung-on-a-chip or what is known as the ‘breathing lung’ that was developed by researchers at Harvard University [63]. The lung-on-a-chip is composed of two identical microchannels that are separated vertically by a thin and PDMS membrane coated with ECM. It allows the culture of alveolar epithelial cells and vascular endothelial cells on the opposite sides of the membrane to reconstruct the alveolar-capillary interface [63]. Additionally, mechanical stretching of the membrane can be produced by applying vacuum pressure to side chambers, thus mimicking the expansion/contraction of the alveolar epithelium during physiological breathing movements in vivo [63]. Hence, the lung-on-a-chip system provides a physiological complexity lacking in other in vitro cell cultures. Accordingly, they have been successful in modelling disease states and assessing drug efficacy and toxicity prior to introduction into clinical trials [64,65].

This miniature lung-on-a-chip platform provides a unique opportunity to study the pathology and the progression of respiratory diseases including infectious diseases affecting the lung such as COVID-19 as well as evaluate the potential antiviral agents. A recent study by Si and colleagues have shown that this microfluidic organ chip can be used as a preclinical in vitro model to study respiratory viruses such as influenza and SARS-CoV-2 having found that they mimic the clinical features of the human lung responses to these viruses [66]. Additionally, they can offer a new in vitro approach to identify new potential therapeutics for these pandemic respiratory viruses [66].

#### 2.2.8. 3D Bioprinting

With the increased emergence of new viral respiratory pathogens and the devastating impacts they have, especially with the latest SARS-CoV-2 pandemic claiming the lives of millions of worldwide, there is an urgent need to fabricate biomimetic respiratory tissue models that recapitulate the human lung microenvironment. Meanwhile, advances in 3D bioprinting have attracted the attention for enabling the production of these tissue models in vitro.

Today, 3D bioprinting technology utilizes computerized methods for manufacturing layers of tissue-like structures by simultaneously yet precisely depositing cells, biological materials and the supportive matrix in layers. This technique is classified into three categories: extrusion- based, laser- based, and inkjet-based printing processes which differ in their precision of cell deposition and the resulting viability of the deposited cells [67,68].

Three-dimensional (3D) bioprinting has been proven to have a wide range of applications including tissue engineering, regeneration, transplantation, drug profiling and screening, and malignant studies [69,70,71]. Moreover, it has been utilized to unravel the pathogenesis of human respiratory viral infections and to study candidate drugs. A study by *Berg* et al. has shown that 3D lung models generated using 3D bioprinting technology supported infection with influenza A virus in a manner similar to that observed in the human lung tissue but not to conventional 2D culture systems [72]. Herein, the study demonstrates the suitability and superiority of the 3D printed lung model over 2D cultures for infection experiments such as influenza [72]. Another recent study by a research team from South Korea was conducted to fabricate a 3D alveolar barrier model by a 3D bioprinting technique using four human alveolar cell lines [73]. The model was successful in simulating the natural human alveoli structurally and functionally. When the alveolar barrier model was infected with respiratory pathogens such as influenza A virus, the viral infectivity and anti-viral responses were observed similarly to the actual tissue [73]. These findings suggest that 3D bioprinting technology can be utilized to develop 3D in vitro lung models that can be used to study SARS-CoV-2 infection and test possible antiviral drugs. Although we are still far from being able to bioprint fully functional organs, this technology is advancing steadily. For instance, Grigoryan et al. 3D bioprinted a ‘vascularized breathing lung model’ using complex technologies in an attempt to engineer a clinically relevant lung tissues [74]. Despite the advantages of these 3D bioprinting techniques, their use has been limited by the requirement of complex and expensive technologies.

Herein, Table 1 summarizes the advantages and disadvantages of different types of 3D scaffold-free and scaffold-based techniques.

## 3. 3D Models for Respiratory Viruses

In vitro studies of human respiratory viral infections were originally conducted in traditional 2D monolayer cell cultures, while in vivo research relied on animal models in an attempt to understand and extrapolate the impact of infection into humans. Nonetheless, these models have limited or different physiological and pathophysiological properties which restrict their ability to mimic the in vivo human microenvironment [79,80]. This necessitates the establishment of in vitro 3D cultures that can provide not only a precise representation of human in vivo tissue structure, environment, proliferation and development, but also accurately portray viral tissue tropism, kinetics, disease pathogenesis and host-pathogen interactions [79,80,81,82]. These 3D models also allow efficient screening of antiviral drugs and testing of the efficacy and safety of vaccines, in addition to investigating the induced immune reaction in response to infection [30,83]. Moreover, 3D cultures can predict the pathogenicity of emergent viruses and serve as a risk assessment tool for future pandemics [80].

### 3.1. Influenza Virus

Influenza viruses are among the most frequently evolving viruses which can infect a wide variety of avian and swine hosts and cross the species obstacles ultimately reaching humans [84]. This presents a huge threat to the global health especially with the previous incidents of avian and swine influenza outbreaks which led to high mortality rates [85,86]. In spite of the advancement reached in cellular biology and virology, it is still challenging to produce an in vitro model to forecast the danger of newly emerging strains. Nonetheless, 3D aggregates of airway respiratory cells seem to provide promising results while being more accessible than other models like bronchus explant culture and more accurate and reliable than the traditional 2D monolayers [79,80]. In a comparative study, Hui et al. was able to prove that airway organoids can replace ex vivo models in influenza studies [87]. These organoids were produced from human lung stem cells and successfully differentiated to goblet, club and epithelial cells with numerous active ciliary structures. When distinctly infected with various strains of human and avian influenza A viruses, the organoids and the ex vivo bronchus explants both showed similar viral titers for each strain. Moreover, susceptibility to each of the viral strains differed between the basal and ciliated epithelial cells, whether in the organoid or tissue explant models [87]. However, this viral tropism was similar in the two models, where in both cell cultures, basal cells were infected by all strains while ciliated cells had higher susceptibility to H1N1 and H7N9 viruses [87]. Moreover, a cytokine assay confirmed the results of the viral kinetics, whereby strains of higher viral titer produced more cytokines. These results were similar in the organoids and the bronchus explants and were reflective of viral infection in vivo, where the higher the pathogenicity of the strain the more severe pro-inflammatory cytokine reaction is [87]. These results allow researchers to replace the tissue explants with organoids which properly recapitulate viral pathogenesis and tropism. In another study, Zhou and colleagues were able to assess the infectivity of several emerging avian and swine influenza strains in the human species and evaluate their potential to cause epidemics [88]. The generated adult stem cells (ASC) organoids showed remarkable differentiation into ciliated, goblet, basal and club cells, where ciliated cells were the most prominent as in the in vivo human airway epithelium. Additionally, these organoids expressed higher levels of serine proteases which is vital for the activation and replication of the influenza A virus [88]. Upon infection, the organoids enhanced the propagation of the human-infective strains in a time range similar to that found in vivo while suppressing the replication of the strains that lack the required human adaptation markers. This study shows that not only do organoids properly recapitulate the morphology and function of in vivo lung tissues, but can also predict the potential of inter-species transmissible influenza strains of causing outbreaks [88]. Murine 3D cultures are also used to study influenza pathogenicity. Quantius et al. used 3D models of mouse epithelial progenitor cells, which upon infection exhibited restricted tissue repair and renewal ability due to the impairment of the β-catenin-dependent Fgfr2b signaling [89]. Treatment of these cells with Fgf10 indorsed the renewal of the epithelial cells after tissue injury caused by influenza infection. These results show how influenza induces and maintains lung epithelium injury and that Fgf10 can be a promising remedy for tissue repair after severe influenza infection [89]. The immunopathology of influenza was also tested by Bhowmick et al. who formed 3D cultures of primary human small airway epithelial cells (HSAEpCs) by seeding the cells on an air–liquid interface (ALI) matrix [90]. Infecting the HSAEpCs 3D cultures with either of the two viral strains H1N1 or H3N2, proved that 3D cultures are a more physiologically relevant model than monolayers whereby the H1N1 strain caused nuclear enlargement and the formation of inclusion bodies in infected HSAEpCs cells which are the same features observed in infected-lung dissection samples [90]. Moreover, H1N1 infection of the 3D cultures revealed severe reductions in alveolar type II protein markers which were observed in previous studies using mice models. This decrease was more severe than that in the H3N2 infected 3D culture which validates previous studies that H1N1 is more immunogenic than H3N2 [90]. Additionally, cytokine profiling of HSAEpCs post infection revealed an increase in the same cytokines as in ex vivo and animal models after infection [90]. The differences observed in the cytokine production between H1N1- and H3N2-infected HSAEpCs cells were also in accordance with the different clinical presentation of each of the strains. Thus, infection of 3D HSAEpCs models thoroughly mimicked infection observed in in vivo studies [90].

### 3.2. Parainfluenza Virus

Parainfluenza presents a big threat to child wellbeing, whereby it causes one fifth of the global annual deaths in children below the age of five due to a severe lower respiratory tract disease [91,92]. This virus is characterized by mutating and evolving according to its host tissue in order to evade the viral inhibitors and the immune response, thus causing it to specifically fit its host [91,92]. This restricts the study of clinical strains in laboratory cell lines which consequently hindered its research capacity. In a study conducted by Porotto et al., lung organoids from a laboratory embryonic stem cell line were tested for compatibility to propagate a clinical parainfluenza virus 3 strain [93]. This study was based on previous findings showing that human airway epithelium did not alter parainfluenza viral genome and proteins. Indeed, the lung organoids endured successful viral propagation without exerting any selective pressure nor modifying the viral genome [93]. Additionally, the results of this study showed that parainfluenza-infected cells had a significant amount of virus contained within the cells without causing cell shedding or any syncytium formation unlike what is observed in RSV and measles infected organoids [93]. This cytopathic effect of lung organoid viral infection is analogous to the clinical presentation of the disease, where parainfluenza is manifested with croup, pneumonia or bronchiolitis and not airway obstruction [93,94]. As such, the lung organoids can be a reliable study model for parainfluenza pathogenesis. 3D cultures were also used to understand the virus–host interactions of animal parainfluenza viruses such as the bovine parainfluenza 3 virus. MDBK spheroids cultured in 3D rotating wall vessels and infected with bovine parainfluenza 3 virus revealed that virus cultured in spheroids had lower replication rate and lower yield, yet a higher ratio of infectious virus particles than that propagated in monolayers [95]. These findings are equivalent to in vivo bovine parainfluenza 3 viral replication which display higher infectivity and are indicative of the spheroids’ ability to productively propagate viruses which are challenging to propagate in conventional monolayers [95].

Considering the absence of any vaccine or treatment for parainfluenza infections, and the inability of traditional 2D cell cultures and animal models to adequately and comprehensively represent infection in humans, researchers started referring to 3D models to investigate parainfluenza therapeutics. A study conducted by NASA on RSV and parainfluenza infections in 3D human bronchio-epithelial tissue-like assemblies showed that not only were the aggregates more representative of in vivo tissue but are also a promising model to test vaccine candidates [94]. These tissue-like assemblies were generated by rotating wall vessel bioreactors and showed properly differentiated cell layers. The cell assemblies were compromised of ciliated and secretory epithelial cells, basement membrane cell, and mesenchymal cells all of which expressed spatial orientation with distinct apical, lateral and basal surfaces and proper polarization of cellular junctions and adhesion molecules similar to lung tissue [94]. Additionally, considering the epithelial cell’s role in mediating an innate immune response upon contracting a pathogen, control 3D assemblies showed higher rates of mRNA expression of genes involved in immune response than uninfected monolayers. These features indicate the ability of the tissue-like assemblies to accurately represent human lung tissue [94]. In regard to the pathogenicity and immunogenicity of the various parainfluenza strains, the attenuated strains showed limited replication and induced less cytokine secretion than the wild-type virus in the 3D assemblies contrary to the monolayer cultures which showed similar viral titers and cytokine secretion levels between the wild type and attenuated virus trails [94]. These findings were concurring with the phase I clinical trial results which showed that inoculation with attenuated parainfluenza strains caused no inflammatory response which enhances severe disease symptoms upon natural infection. Therefore, these 3D models are reliable to test parainfluenza vaccine safety before progressing to clinical trials [94].

### 3.3. Respiratory Syncytial Virus

**Figure 1:**
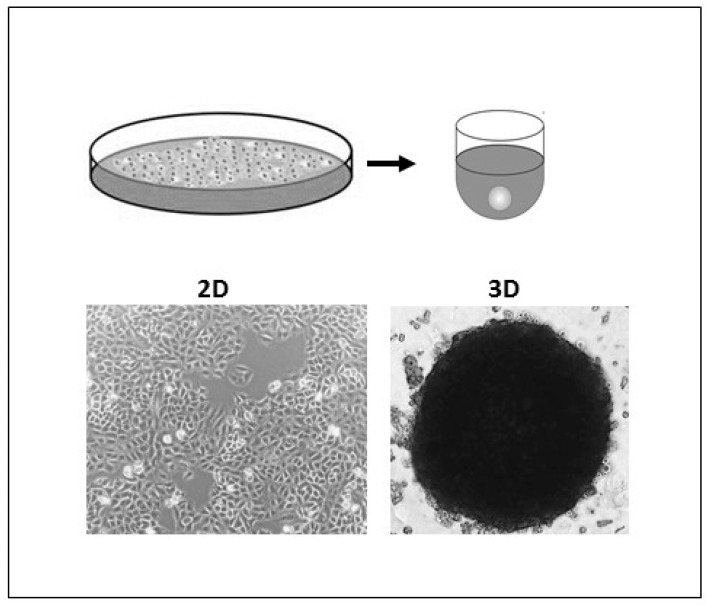
Two-dimensional (2D) and 3D A549 cell cultures. A549 cells were grown as monolayers in 75 cm^2^ plastic tissue-culture flask (left) and as spheroids using ultra-low attachment (ULA) plates to test A549 Spheroid’s permissiveness to Respiratory Syncytial Virus (RSV).

Respiratory Syncytial Virus (RSV) is among the most commonly circulating respiratory tract viruses causing seasonal epidemics. It has high global disease burden where it causes up to 199,000 mortalities annually among infants below the age of five and adults suffering from chronic diseases and immune-deficiency [96,97]. RSV mainly infects alveolar pneumocytes and the ciliated bronchial and bronchiolar cells of the lower respiratory tract. Viral pathogenesis causes swelling and syncytia formation within the infected lung epithelial cells followed by their detachment which is manifested in aggravated mucus production and airway obstruction [97]. Since conventional monolayers and animal models have various limitations and do not precisely recapitulate RSV infections *in vivo*, many studies investigated whether 3D cultures are fit for RSV studies and utilized 3D aggregates and airway organoids to investigate the RSV pathogenesis, therapeutics and the RSV-mediated immune response. In one study, lung bud organoids formed of human pluripotent stem cells (PSCs) were used to test their capacity to recapitulate human lung infections [98]. The cells forming the organoids differentiated into pulmonary mesoderm and endoderm when in culture and demonstrated the same disease features upon their infection as in the lower respiratory tract infections in vivo [98]. Mesenchymal cells expressed the same markers as they would during in vivo infection and showed swelling and sloughing into the organoid lumen [98]. These results demonstrated lung bud organoids’ capacity to reiterate the morphological features of RSV in vivo infection. Another study conducted in our lab demonstrated that 3D spheroids of alveolar epithelial type II cells (A549) were permissive for RSV and allowed its propagation [47]. Similarly to the lung bud organoids mentioned earlier, the infected spheroids demonstrated disease features of clinical infection, where infected cells fused to form large syncytia and had excessive mucin production which accounts for the pulmonary obstruction observed upon human infection [47]. These findings showed that A549 spheroids (Figure 1) are a favorable in vitro model for RSV studies.

Co-cultures of human primary mesenchymal bronchial-tracheal cells and human bronchial epithelial cancerous cells were obtained through rotating wall vessel bioreactors and were tested for their ability to propagate RSV [99]. These culture conditions generated large differentiated spheroids of well-defined tight junctions and desmosomes, and definite polarization and microvilli extrusions. The cells also produced significant amount of mucin and expressed cell markers definitive of cellular differentiation such as villin and keratins [99]. These advanced features of the tissue-like assemblies resemble the properties of true tissue explants and explain the efficient viral replication within these 3D cultures upon inoculation. Moreover, electronic microscopy images showed a time-dependent increase in RSV viral titers and in the build-up of the viral transmembrane glycoproteins indicating efficient viral replication and translation of the viral proteins [99]. Thus, these tissue-like assemblies represented a robust in vitro model for investigating RSV- host tissue interactions and propagation.

Just like parainfluenza, RSV lacks a reliable study model and has no approved vaccine or treatment. As such, 3D aggregates and organoids were used to study RSV therapeutics. The EpiAirway 3D culture model was infected with various RSV strains and treated with various antivirals at different time points [100]. This model indicated which antiviral compounds are the most effective in virus neutralization and indicated that earlier treatment provides better outcomes [100]. A cytokine assay of the infected 3D cultures also revealed an elevation in IL-6, IP-10, and RANTES production which is usually observed upon RSV infection [100]. Another study testing the efficiency of a number of antivirals on 3D human airway epithelium cells (HuAECs) infected with RSV provided evidence on the physiological relevance of the 3D cultures in understanding RSV therapeutics [101]. Finally, the NASA study on parainfluenza infections in 3D human bronchio-epithelial tissue-like assemblies mentioned earlier, also tested the safety of several RSV attenuated viruses as vaccine candidates [94]. Similar results were obtained for the RSV trials as in parainfluenza trials, whereby the 3D assemblies revealed the limited replication and immunogenic capacity of the attenuated viral strains in contrast to the wild type ones [94]. Thus, indicating the relevance of the 3D tissue-like assemblies to serve as a model for screening RSV vaccine candidates.

### 3.4. Coronaviruses

After the first SARS infection was detected in China in 2002, researchers rushed to find a suitable study model for SARS-CoV, since cell lines and animal models did not provide a full understanding of the virus. As such, 3D tissue-like aggregates were developed by co-culturing human bronchial-tracheal mesenchymal cells and human bronchial epithelial cells in the RWV bioreactors [102]. These 3D aggregates were then infected with SARS-CoV to test their permissiveness and susceptibility to the virus while being in the dynamic and highly perfused environment offered by the RWV bioreactors [102]. The aggregates demonstrated higher levels of differentiation than 2D bronchial epithelial monolayers as they expressed more cytoskeletal proteins, mucin, collagen and adhesion molecules and had extensive microvilli on their apical surface. Additionally, cells cultured in aggregates stayed viable for thrice the time as monolayers [102]. Although infection of the 3D aggregates did not yield virus titers, microscopic imaging showed that these cells displayed increased cytoplasmic vacuoles and disrupted endoplasmic reticulum. In addition to that, an intense antigen-antibody reaction was recorded when the cells were treated with polyclonal antibodies against the viral spike and nucleocapsid proteins, in addition to a reaction caused by treatment with an antibody against Group 2 coronavirus [102]. These results indicate that SARS-CoV was able to penetrate the cells and even induce viral protein replication and translation, thus verifying the model’s fitness for coronavirus studies.

Most of our understanding of the infection process is from studies conducted on 2D cultures, which might be misleading as it does not replicate the in vivo microenvironment. Recently, virologists are increasingly using 3D culture models to study the interaction between the virus and the host cell. A study by Milewska and colleagues utilized 3D culture of human airway epithelium (HAE) to map the human coronavirus NL63 (HCoV-NL63) entry pathways [103]. It is also worth noting that the HAE ALI cultures showed so far to be the sole in vitro system for propagating human HCoV-HKU1 coronavirus strain which failed to propagate on all laboratory cell lines [104].

### 3.5. SARS-CoV-2

Since the outbreak of the COVID-19 pandemic, researchers around the world are leaving no stone unturned in the hunt for effective treatments and vaccines to combat the new coronavirus SARS-CoV-2. To achieve their goal, scientists have to rely on effective models that allow studying the disease pathogenesis, understanding virus-host interactions and testing new therapeutics or vaccines. One of the emerging models used in SARS-CoV-2 research is 3D cell cultures models [105,106,107].

As such, Dr. Shuibing Chen and collaborators have generated human pluripotent stem cell-derived lung and colonic organoids (hPSC-LOs and hPSC-COs) using 3D cultures to better understand the disease mechanism of COVID-19 and identify candidate dugs that can block SARS-CoV-2 entry [23]. In their recent publication in Nature, they have shown that lung organoids were permissive to SARS-CoV-2 as expected as the virus primarily targets the respiratory tract, accompanied by robust production in chemokines, which is very similar to what is seen in vivo [23]. As patients with COVID-19 may also experience gastrointestinal symptoms, the response of colonic organoids (hPSC-COs) to SARS-CoV-2 infection was also explored. It was demonstrated that several colonic cell types in hPSC-COs, especially enterocytes, expressed ACE2 (the receptor for SARS-CoV-2), thus are permissive to the virus [23]. The human 3D models were then used to screen for a library FDA-approved drugs. Several drugs, including Imatinib, mycophenolic acid, and quinacrine dihydrochloride were shown to inhibit SARS-CoV-2 entry into both lung and colonic organoids [23].

Moreover, a German research team employed human intestinal organoids derived from pluripotent stem cells (PSC-HIOs) as a tool to study SARS-CoV-2 pathogenesis and its inhibition by Remdesivir, the first FDA-approved drug to treat COVID-19 [108]. Notably, most cell types of the intestinal organoids got infected with SARS-CoV-2 with the exception of goblet cells as they lacked ACE2 expression [108]. More importantly, Remdesivir decreased SARS-CoV-2 infection of intestinal organoids by 86% at a concentration of 0.5 μM and almost inhibited infection at 5 μM, thus indicating Remdesivir as a treatment for SARS-CoV-2 infection of the gut [108]. Another work by Mulay et al. (2021), published in Cell Reports, developed a 3D model of human alveoli, alveospheres, by growing human alveolar type 2 (AT2) cells in Matrigel and then studied how SARS-CoV-2 affects it [109]. It was found that alveospheres were readily infected by SARS-CoV-2, with expression of the SARS-CoV-2 entry receptor, ACE2, on AT2 cells. This was associated with an inflammatory response and upregulation of the interferon signaling pathway [109]. Similarly, two other studies using primary lung alveolar organoids for the study of SARS-CoV-2, reported induction of interferon response infections [107,110]. On the other hand, Ramani et al. utilized iPSCs-derived 3D human brain organoids as a model system to test the neurotoxic effects of SARS-CoV-2 [111]. It was shown that the virus targets the neurons of 3D brain organoids despite a low level of ACE2 expression. This finding has multiple explanations, either a basal level of ACE2 is sufficient for SARS-CoV-2 entry into neurons or the virus enters via ACE2-independent pathways [111].

Together, these data demonstrate that 3D cultures can serve as reliable in vitro human models to study SARS-CoV-2 infection and provide a relevant platform for drug screening to identify candidate COVID-19 therapeutics.

## Figures and Tables

**Table 1:** Three-dimensional spheroid-generating systems.

	Culture Model	Advantages	Disadvantages	References
**Scaffold based methods**	Hydrogels 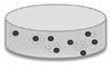	- Hydrophilic nature - Environment mimics ECM - Matrices can be adjusted to fit research purpose - Biodegradable/Non-biodegradable - Proper of a wide range of cell types	- Labor intensive - Long production time - Variability in composition between batches - Biodegradable/Non-biodegradable	[16,18]
**Non-scaffold based methods**	Bioreactors 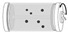	- Enhanced natural diffusion of gas and nutrients in spheroids - Proper for primary cells, cell lines and co-cultures	- Inconsistent spheroid size - Difficult to visualize and monitor	[26,30]
	Spinner flasks 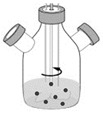	- Enhanced natural diffusion of gas and nutrients in spheroids - Proper for primary cells, cell lines and co-cultures - Large yield of spheroids	- Difficult to visualize and monitor - Causes harmful shear stress forces - Not suitable for cell types which fail to survive and assemble in suspensions	[33,34]
	Hanging drops 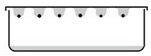	- Proper for primary cells, cell lines and co-cultures - Easy to monitor - Require only basic laboratory equipment	- Limited yield - Labor intensive	[35,36]
	Ultra-low attachment plates 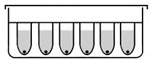	- Affordable - Simple and easy - Reproducible and consistent spheroid size and shape - Compatible for high throughput screening - Easy to monitor	- Limited access to media in a part of the spheroid - Limited yield - Incompatible for large spheroids	[45,46,47]
	Centrifugation pellet cultures 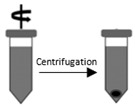	- Easy and simple for use - Large yield of spheroids	- Causes harmful shear stress forces - Difficult to visualize and monitor - Generated spheroids are of large girth	[75,76]
	Electric, Magnetic, and Ultrasound based cultures 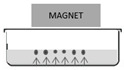	- Strictly managed settings	- Limited ability to regulate the spheroids’ size - Inconvenient and requires specific equipment - The external forces can alter the physiology of the cells	[50,51,75,77]
	Microwell arrays 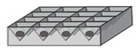	- Easy and simple for use - Reproducible and consistent spheroid size and shape - Generation of complex-shaped spheroids - Economical - Compatible for use in technologically modest laboratories. - Compatible for high throughput screening and standard cell culture monitoring techniques	- Incompatible for large spheroids	[52,53]
	Microfluidics 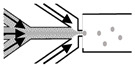	- Large yield of spheroids - Consistent spheroid size - Easy and simple for use - Fast production rate - Compatible for high throughput screening - Proper for primary cells, cell lines and co-cultures - Enhanced natural diffusion of gas and nutrients in spheroids - High oxygen concentrations promoting cell viability	- Limited to technologically advances laboratories - Limited yield	[24,59,78]
	3D bioprinting 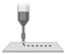	- Accurately arranges the cells - Enhances cell viability, function, migration and self-assembly - Compatible for high throughput screening	- Cell viability varies based on cross-linking and the shear stress of passing through the nozzle - Expensive material of limited availability - Long maturation time	[68,69,70]

## Data Availability

Not applicable.
